# Incidence and predictors of complications in Gram-negative bloodstream infection

**DOI:** 10.1007/s15010-024-02202-3

**Published:** 2024-03-04

**Authors:** Utpal Mondal, Erin Warren, P. Brandon Bookstaver, Joseph Kohn, Majdi N. Al-Hasan

**Affiliations:** 1 Department of Medicine, Division of Infectious Diseases, Audie L. Murphy VA Medical Center, San Antonio, TX USA; 2 Department of Medicine, Long School of Medicine, University of Texas Health Science Center, San Antonio, TX USA; 3 Department of Pharmacy, Prisma Health-Midlands, Columbia, SC USA; 4 Department of Clinical Pharmacy and Outcomes Science, University of South Carolina College of Pharmacy, Columbia, SC USA; 5 Department of Medicine, University of South Carolina School of Medicine, Columbia, SC USA; 6 Department of Internal Medicine, Division of Infectious Diseases, Prisma Health-Midlands, Columbia, SC USA

**Keywords:** Bacteremia, Sepsis, Infective endocarditis, Endovascular infection, Discitis, Epidural abscess

## Abstract

**Background:**

The incidence of metastatic complications in Gram-negative bloodstream infection (GN-BSI) remains undefined. This retrospective cohort study examines the incidence and predictors of complications within 90 days of GN-BSI.

**Methods:**

Patients with GN-BSIs hospitalized at two Prisma Health-Midlands hospitals in Columbia, South Carolina, USA from 1 January 2012 through 30 June 2015 were included. Complications of GN-BSI included endocarditis, septic arthritis, osteomyelitis, spinal infections, deep-seated abscesses, and recurrent GN-BSI. Kaplan–Meier analysis and multivariate Cox proportional hazards regression were used to examine incidence and risk factors of complications, respectively.

**Results:**

Among 752 patients with GN-BSI, median age was 66 years and 380 (50.5%) were women. The urinary tract was the most common source of GN-BSI (378; 50.3%) and *Escherichia coli* was the most common bacteria (375; 49.9%). Overall, 13.9% of patients developed complications within 90 days of GN-BSI. The median time to identification of these complications was 5.2 days from initial GN-BSI. Independent risk factors for complications were presence of indwelling prosthetic material (hazards ratio [HR] 1.73, 95% confidence intervals [CI] 1.08–2.78), injection drug use (HR 6.84, 95% CI 1.63–28.74), non-urinary source (HR 1.98, 95% CI 1.18–3.23), BSI due to *S. marcescens, P. mirabilis* or *P. aeruginosa* (HR 1.78, 95% CI 1.05–3.03), early clinical failure criteria (HR 1.19 per point, 95% CI 1.03–1.36), and persistent GN-BSI (HR 2.97, 95% CI 1.26–6.99).

**Conclusions:**

Complications of GN-BSI are relatively common and may be predicted based on initial clinical response to antimicrobial therapy, follow-up blood culture results, and other host and microbiological factors.

## Introduction

An estimated 500,000 individuals develop bloodstream infections (BSI) every year in the USA [1]. The incidence and the predictors of complications in Gram-positive BSI are well documented in previous literature. For instance, the incidence of complications in *Staphylococcus aureus* BSI is up to 40% [2]. There are robust models that predict complications in *S*. *aureus*, *Streptococcus* species, and *Enterococcus* species BSI [2–6]. There is a paucity of evidence-based data on the incidence and risk factors of complications in Gram-negative BSI (GN-BSI).

Despite early identification of Gram-negative bloodstream isolates using rapid diagnostics and subsequent improvements in antimicrobial management, case–fatality rate of GN-BSI remains as high as 13–15% [1, 7, 8]. Early identification of complications in patients with GN-BSI could lead to prompt and adequate source control and improved outcomes.

This retrospective cohort study determined the incidence of complications within 90 days of GN-BSI and examined clinical and microbiological risk factors for these complications.

## Patients and methods

### Settings

The study was performed at Prisma Health Richland and Baptist Hospitals in Columbia, South Carolina, USA. The two community-teaching hospitals combine for over 1000 licensed beds and provide medical, surgical, and subspecialty care for the residents of Richland and surrounding counties in the Midlands region of South Carolina, USA. The Institutional Review Board at Prisma Health approved the study and waived informed consent.

### Definitions

GN-BSI was defined as growth of aerobic or facultative anaerobic Gram-negative bacilli in a blood culture. Blood cultures with mixed growth were excluded. The Charlson comorbidity and Pitt bacteremia scores were used to assess chronic comorbidities and acute severity of illness at the onset of GN-BSI, respectively [9, 10]. Initial response to antimicrobial therapy within 72–96 h of GN-BSI was evaluated using the early clinical failure criteria (ECFC) [11].

Septic arthritis, osteomyelitis, spinal infections, deep-seated abscesses, infective endocarditis, other endovascular infections (e.g., mycotic aneurysms), and recurrent GN-BSI within 90 days of the initial episode of GN-BSI were considered as potential complications [2]. Septic arthritis and osteomyelitis, in the presence or absence of hardware, were defined based on clinical symptoms and signs of infection in the presence of radiographic evidence, and/or the same Gram-negative bloodstream isolate in the joint fluid or bone, respectively. Spinal infections included discitis, vertebral osteomyelitis, and epidural and paraspinal abscesses and were defined based on clinical and radiographic criteria in the presence of the same Gram-negative bloodstream isolate in the respective compartment. Deep-seated abscesses included musculoskeletal, perinephric, liver, and peritoneal abscesses. Growth of the same Gram-negative bloodstream isolates in the abscess fluid was required for diagnosis. Infective endocarditis was defined per the modified Duke criteria and included native, prosthetic valve, pacemaker, and intracardiac device endocarditis [12]. Recurrent GN-BSI was defined as subsequent GN-BSI due to the same genus and species of the Gram-negative bacteria within 90 days of the initial episode of GN-BSI. The presence of prosthetic material was defined as indwelling central venous catheters (dialysis catheters, intravenous ports, non-tunneled catheters, etc.), endovascular grafts, prosthetic heart valves, intracardiac devices, prosthetic joints, or other orthopedic or spinal hardware at the time of index GN-BSI. Persistent bacteremia was defined as growth of the same Gram-negative bacteria on repeat blood cultures obtained between 24 and 96 h of the index GN-BSI.

### Case ascertainment

In this retrospective cohort study, all hospitalized patients ≥ 18 years old with mono-microbial GN-BSI from 1 January 2012 through 30 June 2015 were identified through the microbiology laboratory databases at the two Prisma Health-Midlands Hospitals. Adult patients with first episodes of GN-BSI admitted to any medical, surgical, or intensive care unit during the study period were included in this cohort as described elsewhere [7].

### Statistical analysis

The primary objectives of this retrospective observational cohort study were to examine incidence and risk factors of complications within 90 days of index GN-BSI. Kaplan–Meier analysis was used to examine the incidence of complicated GN-BSI. Patients were followed up for 90 days from index GN-BSI or until detection of complications, death, or loss to follow-up. This allowed censoring of patients who died or were lost to follow-up within 90 days of GN-BSI. A Univariate Cox proportional hazards regression model was used to examine risk factors for complications. Variables associated with complications in univariate analysis (*p* < 0.05) were included in the multivariate Cox proportional hazard regression model using backward selection. Hazard ratios (HR) and 95% confidence intervals (CI) were used to characterize the associations of each risk factor with complications of GN-BSI. JMP v.16.0 (SAS Institute Inc., Cary, NC) was used for statistical analysis. The level of statistical significance was defined as a two-sided *p* value < 0.05 unless otherwise specified.

## Results

A total of 752 patients with GN-BSI were included in the study. The median age was 66 years and 380 (50.5%) were women. The baseline demographics and the clinical characteristics of the cohort are shown in Table 1. The urinary tract was the most common source of GN-BSI (378; 50.3%), followed by central venous catheter infection (66; 8.8%), gastrointestinal tract (52; 6.9%), biliary tract (42; 5.6%), respiratory tract (40; 5.3%), skin and soft tissue infection (33; 4.4%), and other sources (12; 1.6%). The remaining 129 (17.2%) patients had unknown source of infection. *Escherichia coli* was the most common bacteria (375; 49.9%), followed by *Klebsiella* species (158; 21.0%), *Enterobacter* species (53; 7.0%), *Proteus mirabilis* (46; 6.1%), *Pseudomonas aeruginosa* (42; 5.6%), *Serratia marcescens* (25; 3.3%), and other Gram-negative bacteria (53; 7.0%).

**Table 1:** Baseline demographics and clinical characteristics of patients with Gram-negative bloodstream infection

Variable	*N* = 752
Age in years, median (IQR)	66 (55–77)
Female, *n* (%)	380 (50.5)
Race, *n* (%)	
African American	377 (50.1)
White	346 (46.0)
Other	29 (3.9)
Diabetes mellitus, *n* (%)	298 (39.6)
End-stage renal disease, *n* (%)	80 (10.6)
Liver cirrhosis, *n* (%)	42 (5.6)
Cancer, *n* (%)	124 (16.5)
Immune compromised host, *n* (%)	85 (11.3)
Indwelling prosthetic material, *n* (%)	252 (33.6)
Injection drug use, *n* (%)	6 (0.8)
Community-onset acquisition, *n* (%)	568 (75.5)
Charlson comorbidity score, median (IQR)	2 (0–3)
Pitt bacteremia score, median (IQR)	2 (1–3)
Inappropriate empirical antimicrobial therapy, *n* (%)	51 (6.8)
Early clinical failure criteria, median (IQR)	1 (0–2)

IQR: interquartile range

Overall, 79 patients developed complications within 90 days of index GN-BSI. Deep-seated abscesses, bone/joint infections, endovascular infections, and recurrent GN-BSI constituted the four major complication categories (Table 2). The median time to identification of complications was 5.2 days from the index GN-BSI (interquartile range 1–28 days). The overall incidence of complications in patients with GN-BSI was 13.9% in the Kaplan–Meier analysis. Complications were significantly more common in BSI due to *Serratia marcescens* (39.7%)*, Proteus mirabilis* (35.7%), and *Pseudomonas aeruginosa* (21.5%) than other GN-BSI (11.1%; log-rank *p* < 0.001).

**Table 2:** Complicated Gram-negative bloodstream infection

Complication	*N* = 79
Deep-seated abscess	26 (32.9)
Liver abscess	10 (12.7)
Peritoneal abscess	6 (7.6)
Retroperitoneal abscess (perinephric, etc.)	6 (7.6)
Musculoskeletal abscess (iliopsoas, etc.)	4 (5.1)
Bone, joint, and spinal infection	20 (25.3)
Osteomyelitis	8 (10.1)
Septic arthritis	8 (10.1)
Spinal infection (discitis, epidural abscess, etc.)	4 (5.1)
Endovascular infection	18 (22.8)
Infective endocarditis	11 (13.9)
Other vascular infection (vascular graft, etc.)	7 (8.9)
Recurrent Gram-negative bloodstream infection	15 (19.0)

Data are shown as number (%)

**Fig. 1:**
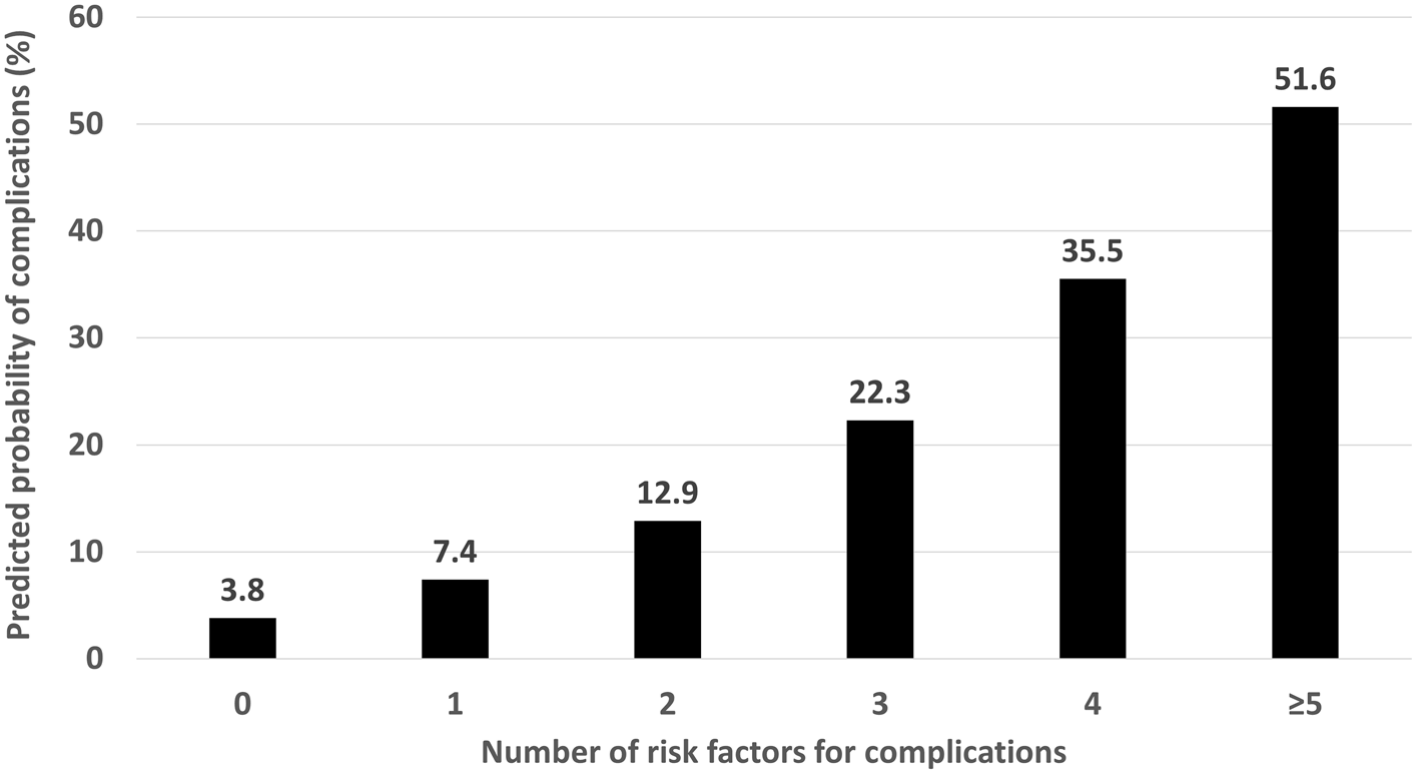
Predicted risk of complications in Gram-negative bloodstream infection based on number of risk factors. Risk factors for complications are presence of prosthetic material, injection drug use, non-urinary source of infection, bloodstream infection due to *Serratia marcescens*, *Pseudomonas aeruginosa*, or *Proteus mirabilis*, delayed clinical response to therapy within first 72 h (early clinical failure criteria ≥ 2), and persistent bacteremia

In the univariate Cox proportional hazards regression model, end-stage renal disease, presence of indwelling prosthetic material, injection drug use, non-urinary source of infection, BSI due to *S. marcescens*, *P. mirabilis*, or *P. aeruginosa*, ECFC, and persistent GN-BSI were associated with higher risk of complications (Table 3). After adjustments in the multivariable Cox model, end-stage renal disease was not an independent risk factor for complications (HR 0.87, 95% CI 0.43–1.75, *p* = 0.70). Predictors of complications were indwelling prosthetic material, injection drug use, non-urinary source, BSI due to *S. marcescens*, *P. mirabilis*, or *P. aeruginosa*, ECFC, and persistent GN-BSI (Table 4)**.** The predicted probability of complications increased from 3.8% in the absence of risk factors to 51.6% in patients with ≥ 5 risk factors (Fig. 1).

**Table 3:** Univariate Cox proportional hazards regression results for potential risk factors for complicated Gram-negative bloodstream infection

Risk factor	HR (95% CI)	*P*-value
Age per decade	0.94 (0.83–1.07)	0.33
Female sex	0.82 (0.53–1.28)	0.39
White race	1.12 (0.72–1.74)	0.62
Diabetes mellitus	1.19 (0.77–1.86)	0.43
End-stage renal disease	2.00 (1.14–3.51)	0.02
Liver cirrhosis	0.68 (0.21–2.14)	0.51
Cancer	1.02 (0.55–1.89)	0.95
Immune compromised host	0.60 (0.24–1.49)	0.27
Indwelling prosthetic material	2.02 (1.30–3.15)	0.002
Injection drug use	4.92 (1.20–20.17)	0.03
Community-onset acquisition	0.75 (0.46–1.23)	0.25
Charlson comorbidity score (per point)	0.97 (0.85–1.08)	0.56
Non-urinary source of BSI	2.50 (1.55–4.01)	< 0.001
*S. marcescens*, *P. aeruginosa*, or *P. mirabilis* BSI	2.51 (1.54–4.08)	< 0.001
Pitt bacteremia score (per point)	1.05 (0.96–1.14)	0.27
Inappropriate empirical antimicrobial therapy	1.41 (0.69–2.85)	0.34
Early clinical failure criteria (per point)	1.25 (1.09–1.44)	0.002
Persistent BSI	4.14 (1.80–9.52)	< 0.001

HR: hazards ratio; CI: confidence intervals; BSI: bloodstream infection

**Table 4:** Multivariate Cox model results for independent risk factors for complicated Gram-negative bloodstream infection

Risk factor	HR (95% CI)	*P*-value
Indwelling prosthesis	1.73 (1.08–2.78)	0.02
Injection drug use	6.84 (1.63–28.74)	0.008
Non-urinary source of BSI	1.98 (1.18–3.33)	0.009
*S. marcescens*, *P. aeruginosa*, or *P. mirabilis* BSI	1.78 (1.05–3.03)	0.03
Early clinical failure criteria (per point)	1.19 (1.03–1.36)	0.02
Persistent BSI	2.97 (1.26–6.99)	0.01

*HR* hazards ratio, *CI* confidence intervals, *BSI* bloodstream infection

## Discussion

Although GN-BSI is a relatively common cause of morbidity and mortality in the general population [1], incidence and risk factors for complications have not been previously established. In this cohort of patients who were admitted to two community hospitals in southeastern U.S., 13.9% developed complications within 90 days of GN-BSI. Deep-seated abscesses (liver, peritoneal, retroperitoneal, and musculoskeletal) contributed nearly one-third of complications. Bone, joint, and spinal infections combined for one-fourth of complications. Infective endocarditis and other vascular complications combined for another one-fourth. The remaining complications were recurrences.

The study identified BSI due to *P. mirabilis*, *P. aeruginosa*, and *S. marcescens* as an independent risk factor for complications. The prevalence of complications was 22–40% in BSI due to these bacteria as compared to 11% among other Gram-negative bacilli. The current results are, in part, consistent with those of a relatively recent study in North Carolina that demonstrated comparable risk of cardiac device-related infective endocarditis in patients with BSI due to *S. aureus*, *P. aeruginosa*, and *S. marcescens*. [13] This risk was considerably and significantly higher than that of BSI due to other bacteria [13]. Accordingly, the recently updated modified Duke criteria included *P. aeruginosa* and *S. marcescens* in the list of typical bacteria commonly causing endocarditis in individuals with intracardiac prosthetic material [14]. However, the identification of *P. mirabilis* BSI as a risk factor for complications was unexpected. This observation needs further examination in future investigations.

In addition to microbiological risk factors, the study identified multiple host, clinical, and laboratory predictors of complications in GN-BSI. Non-urinary source of GN-BSI, presence of indwelling prosthetic material, and injection drug use were associated with higher risk of complications in this study. This is conceivable in the broader context of previous studies in the field. Non-urinary source of infection has been associated with higher mortality in patients with GN-BSI [15]. It usually implies a high bacterial burden and increased probability of inadequate source control, which may increase the risk of dissemination. The presence of prosthetic material promotes development of biofilms and may increase the risk of dissemination despite appropriate antimicrobial therapy. Injection drug use has been associated with increased risk of complications in patients with BSI due to Gram-positive bacteria [4]. The study population included only a small number of patients with history of injection drug use. It is difficult to make a definitive risk association based on this finding alone. It would be prudent to further investigate injection drug use as a potential risk factor for complications in GN-BSI now that the incidence of injection drug use has increased in various geographical regions in the USA.

The study identified delayed clinical response to antimicrobial therapy as a predictor of complications. The ECFC previously predicted clinical outcomes (mortality, hospital length of stay, and treatment failure) in patients with *Enterococcus* species, *Streptococcus* species, and GN-BSI [11, 16, 17]. The current study demonstrated that ECFC may be used to predict complications in GN-BSI. This is clinically conceivable since persistent tachycardia, hypotension, respiratory failure, altered mental status, or leukocytosis despite 3 days of appropriate antimicrobial therapy should prompt further work-up for detection of complications as potential source control opportunities. Patients had a 19% increased risk of complications by each point increase in ECFC. It would be interesting to examine the utility of ECFC in predicting complications in BSI due to *S. aureus* and other Gram-positive bacteria in future investigations.

The role of follow-up blood cultures in GN-BSI has recently evolved. Multiple recent studies have demonstrated a survival benefit in patients with GN-BSI who had follow-up blood cultures obtained than those who did not have follow-up blood cultures [18–20]. Additionally, in patients who had follow-up blood cultures obtained, persistent GN-BSI was associated with increased mortality [18]. The current study provides another reason to obtain follow-up blood cultures in patients with GN-BSI since persistent GN-BSI was associated with a threefold increase in the risk of complications. This is consistent with the results of a recent study that demonstrated that the persistent detection of Gram-negative bacteria in the blood using culture-independent techniques was associated with increased risk of complications and opportunities for source control [21].

The current study presents a framework for the work-up of potential complications in GN-BSI. The probability of complications is relatively low in patients with < 2 risk factors for complications. It could be argued that no additional work-up is required in those patients in the absence of focal signs or symptoms suggestive of complications. The predicted probability of complications increases to 12.9%, 22.3%, 35.5%, and 51.6% in the presence of 2, 3, 4, and ≥ 5 risk factors, respectively. These patients likely require additional work-up for potential complications. For example, an echocardiogram is indicated in patients with prosthetic cardiac device and *S. marcescens* or *P. aeruginosa* BSI in concordance with the recently modified Duke criteria for infective endocarditis [14]. A similar work-up may be justified in a patient with recent injection drug use and GN-BSI secondary to unknown source of infection. Other imaging studies may be indicated depending on the clinical scenario. In a patient with *P. mirabilis* BSI secondary to a urinary source of infection, presence of ≥ 2 ECFC after 72 h of appropriate antimicrobial therapy may prompt a retroperitoneal ultrasound or non-contrast CT scan to entertain the possibility of a perinephric abscess or a ureteric stone causing obstruction. Alternatively, an MRI of the lumbar spine may be preferred in the same patient if back pain that was initially attributed to acute pyelonephritis failed to improve despite otherwise appropriate management. A CT scan of the abdomen with contrast may be beneficial in a patient with history of aortic vascular graft repair and persistent GN-BSI despite appropriate antimicrobial therapy to rule out the possibility of a vascular graft infection. Threshold, modality, and timing of further work-up for potential complications are implicated by the total number of risk factors, type and location of host factors, and presence of clinical and laboratory risk factors, respectively. A more aggressive work-up for complications and source control may be required in patients with ≥ 3 risk factors given the high probability of complications. The presence of prosthetic material in a specific anatomic location marks that site for potential imaging in the absence of focal symptoms or signs of infection elsewhere. Since ECFC and follow-up blood culture results become available on day 3 of index GN-BSI, it may be the optimal time for work-up of complications in patients who do not already have multiple host and microbiological risk factors for complications at initial presentation. This framework provides an opportunity for earlier detection of complications by at least 2 days as compared to the median of 5 in the current study population.

To our knowledge, this is the first study to establish the incidence and risk factors for complications in GN-BSI. The study shares common limitations of retrospective cohorts, including the possibility of unknown or unmeasured confounders. Work-up and diagnosis of complications in this study were up to the discretion of the primary healthcare provider. A prospective design with a standard clinical protocol or pathway for work-up of complications would provide advantages. The framework provided in the current investigation may facilitate the design of future prospective studies on this topic. At the time of the study, positron emission tomography with computed tomography (PET/CT) was not commonly performed in clinical practice to identify potential complications. As such, it might be plausible that the current study underestimated the incidence of complications. Moreover, the incidence of complications might have been underestimated since many patients who died early after GN-BSI might not have lived long enough to allow imaging or other work-up for complications. Using Kaplan–Meier analysis and censoring of patients at the time of death may have minimized this impact. Finally, the study included patients from two community hospitals in the same healthcare system and geographical location. The characteristics of this cohort resemble the local population of the Midlands region of South Carolina, USA. The incidence of complications may vary in populations representing other ethnic groups, medical complexity, rates of injection drug use, and microbiology. Further investigations in other populations would increase the generalizability of the current findings.

## Conclusion

Complications in GN-BSI are relatively common, particularly in patients with multiple risk factors. The risk of complications may be predicted by host factors, microbiology of BSI, initial clinical response to therapy within 72 h of GN-BSI, and follow-up blood culture results. Stratification of patients based on risk factors may aid in identifying those requiring further diagnostic work-up for early detection of complications and more aggressive approach to source control.

## Data Availability

Deidentified data in this manuscript will be available upon request from the corresponding author after signing a data sharing agreement. Any further statistical analyses should be performed in collaboration with and under the guidance of the corresponding author.
